# Modeling Population Exposure to Ultrafine Particles in a Major Italian Urban Area

**DOI:** 10.3390/ijerph111010641

**Published:** 2014-10-15

**Authors:** Andrea Spinazzè, Andrea Cattaneo, Carlo Peruzzo, Domenico M. Cavallo

**Affiliations:** 1 Department of Science and High Technology, University of Insubria, via Valleggio 11, 22100 Como, Italy; E-Mails: andrea.cattaneo@uninsubria.it (A.C.); domenico.cavallo@uninsubria.it (D.M.C.); 2 Occupational and Preventive Health Division, Macchi Foundation Hospital, Viale Borri 57, 21100 Varese, Italy; E-Mail: carlo.peruzzo@ospedale.varese.it

**Keywords:** ultrafine particles, microenvironments, indoor environments, commuting, employment status, education level, age, gender, general population

## Abstract

Average daily ultrafine particles (UFP) exposure of adult Milan subpopulations (defined on the basis of gender, and then for age, employment or educational status), in different exposure scenarios (typical working day in summer and winter) were simulated using a microenvironmental stochastic simulation model. The basic concept of this kind of model is that time-weighted average exposure is defined as the sum of partial microenvironmental exposures, which are determined by the product of UFP concentration and time spent in each microenvironment. In this work, environmental concentrations were derived from previous experimental studies that were based on microenvironmental measurements in the city of Milan by means of personal or individual monitoring, while time-activity patterns were derived from the EXPOLIS study. A significant difference was observed between the exposures experienced in winter (W: 28,415 pt/cm^3^) and summer (S: 19,558 pt/cm^3^). Furthermore, simulations showed a moderate difference between the total exposures experienced by women (S: 19,363 pt/cm^3^; W: 27,623 pt/cm^3^) and men (S: 18,806 pt/cm^3^; W: 27,897 pt/cm^3^). In addition, differences were found as a function of (I) age, (II) employment status and (III) educational level; accordingly, the highest total exposures resulted for (I) 55–59 years old people, (II) housewives and students and (III) people with higher educational level (more than 10 years of scholarity). Finally, significant differences were found between microenvironment-specific exposures.

## 1. Introduction

Exposure may be defined as the concentration of a particular agent that reaches a target organism, system, or population in a specific frequency for a defined duration [1]. Exposure can also be defined as the contact of a target and a chemical, physical, or biological agent in an environmental carrier medium [2,3,4]. More expansively, exposure denotes the contact between an agent and a target, which takes place at a contact boundary or surface over an exposure period. Exposure to ultrafine particles (UFP, <100 nm) is an important topic in epidemiological and toxicological studies and is deemed to be a major risk affecting human health. Therefore, airborne particle studies were performed in the recent years to identify the main UFP sources and to characterize population exposure. Exposure can be measured or modeled [5], either directly (personal measurements) or indirectly (microenvironment approach) [3,4,5,6,7,8,9,10].

In order to properly evaluate the UFP exposure, personal monitoring is considered as the only way to relate particle exposure levels to the activities performed and microenvironments visited. For example, a recent study carried out in central Italy during summer and winter in 2012 [11] evaluated the influence of time-activity patterns on the personal exposure of 24 Italian couples to UFPs based on their time-activity patterns, through an experimental measurement of personal exposure over 48 h. Time-activity patterns, particle number concentration exposure and the related dose received by the participants (in terms of particle alveolar deposited surface area) were measured. Similarly, in another study [12] the examination of personal behavior and activity was combined with the measurement of particulate matter with high temporal resolution and over full 24 h periods using an optical aerosol spectrometer. Personal monitoring offers the most accurate measurements of exposure to air pollutants. The drawback of such methods, however, is the high cost of implementation and the associated small number of observations that tends to produce sample biases: only specific types of subjects would carry monitors and record their daily activities for a relatively prolonged time period. For this reason, personal monitoring is often used as a complement in exposure models to assess air pollution exposures in health studies. These models use personal or household exposure monitoring, and appear well-suited to overcome the problem of achieving population representative samples while understanding the role of exposure variation at the individual level. Thus, exposure modeling is recognized as a valuable and cost-effective tool for assessing potential population exposures to air pollution and represent an element of exposure assessment, which evaluates, qualitatively and quantitatively, the degree of intake or uptake that is likely to occur. Exposure models allow estimation of pollutant exposure for groups of people and time periods for which personal monitoring has not been conducted; models can be also used to combine information from different sources to produce estimates for population exposures that would be very expensive or impossible to perform [13,14]. For example, some studies reviewed in Jerrett *et al.* [15] combine personal or regional monitoring with other air pollution exposure methods (hybrid models) in order to compare or validate results from exposures assigned from modeling of ambient exposure with the use of experimental monitoring at differing scales (*i.e.*, personal and regional monitoring). These methods appear well-suited to overcome the conundrum of achieving population representative samples while understanding the role of exposure variation at the individual level. Remote sensing and activity–space analysis will complement refinements in pre-existing methods, and permit to reduce scientific uncertainties in exposure analysis. An application of activity–space analysis may be found in a recent study [16], in which activity-pattern data were combined with microenvironmental data (human activities and particle number size distributions) using an indirect approach, in order to evaluate the doses of alveolar and tracheobronchial deposited particle number and surface area experienced by different age groups in south and north Italy. This study used the average particle number size distribution data obtained from an experimental measurement survey in major microenvironments, together with activity pattern data to estimate the tracheobronchial and alveolar dose of submicrometer particles for different population age groups in Italy. Furthermore, time-activity patterns were combined with microenvironmental data through a Monte Carlo simulation in order to evaluate the daily alveolar and tracheobronchial number or surface area deposited doses for different age group populations [17]. More generally, physical stochastic models describe parameters with frequency or probability distributions instead of single values. These models can be used to predict population exposures for existing, past or scenario situations and for subpopulations with no available measurement data [5], by simulations based on the distributions of input parameters. In this case, the full description of personal exposure to an air pollutant requires knowledge of the magnitude of pollutant concentration in the exposure environment, duration and time pattern of exposure [5]. As mentioned before, the microenvironment (ME) approach [18] has been commonly used to model exposures [7,14,19,20,21]. In such a case, the exposure (*E*) is calculated as the sum of the partial exposures across the visited MEs according to the relationship described by Equation 1 (where *C_i_* is the concentration in the *i*th microenvironment, *T_i_* is the fractional time spent in the *i*th microenvironment, and *N* is the number of microenvironments). The exposure *E* is often defined as “total exposure”, but this study refers to the term “time-weighted average exposure”, because the simulated exposure *E* is the total exposure (expressed in particle/cm^3^) for the considered subpopulation, expressed as the average concentrations weighted on the integration period (24 h).

(1) $$E=\sum_{i}^{N}C_{i}T_{i}$$

This paper describes the simulation of exposure to UFP and evaluates the differences of the estimates by subpopulation and season. A microenvironmental probabilistic exposure model was developed in order to simulate the exposure of different subpopulations to UFP in the city of Milan, distinguished by gender, age, employment status and educational level. Our approach includes the use of time-activity data of subpopulations within the study area and average concentrations in different environments collected by on-site experimental measurements. The present study was carried out in the city of Milan, which is the second largest city in Italy and has a population of more than one million inhabitants. Its urban area (181 km^2^) is characterized by a high density of residential and commercial buildings and very high traffic volumes, while many factories are located at the city’s outskirts. As with many large cities, Milan suffers from high levels of air pollution, especially in winter, during which time air quality limits are frequently exceeded and exceptionally high particulate matter (PM) mass concentrations are frequently recorded [22]. The UFP concentrations are usually particularly high along busy roads, common in urban transport environments [23,24,25], generated in large quantities by fuel combustion processes, with vehicular traffic exhaust being the predominant source in urban environments [26]. The main objective of this study was to estimate individual UFP exposures in general subpopulations during a typical weekday for summer and winter periods within the metropolitan area of Milan (Italy). The quantification of daily exposures for the general population is important to provide better estimates in investigations of long-term health effects. Other specific aims are (I) to use the model to simulate the daily mean exposures to UFP and (II) to observe exposure distributions and differences among different subpopulations as a function of seasonal variability (summer and winter) and behavioral factors (time use).

## 2. Experimental Section

Exposure models based on Equation (1) should describe the microenvironmental concentration of the considered pollutant and fully characterize the behavior (time use) of the study population. Relevant microenvironments need to be defined to perform exposure simulation; at the same time, data on time-activity patterns are needed, specified as the amount of time spent in each microenvironment. People spend their time differently, depending for example on employment status, age [14], season, and day of week [27]. Exposure models require data on human time patterns: time-activity data are required implicitly to determine the status of source use, the activity level of subjects, and other activities that may affect exposure components. Therefore, it is important to define groups of people with similar time-activity patterns. The exposure distributions for subpopulations need to be simulated separately, and eventually merged together to get an exposure distribution for the overall population.

### 2.1. Input Data: Time Activity Patterns

The present study focused on the city of Milan; the subpopulations were firstly defined on the basis of gender, and then for age, employment status or educational level (Table 1). Subpopulations were defined on the basis of expected general similarity of time-activity patterns within groups. This selection of subpopulations and MEs was also made in accordance with the availability of activity pattern data: the selected source was the EXPOLIS study, in which the time (mean, standard deviation) spent in 11 different MEs (Table 2) by Milan’s subpopulation (years: 1996–2000) was described and whose results are available online [28]. Time-activity data refer to the typical working day (excluding weekend and holiday) without seasonal distinction and allow defining the amount of time spent daily in each microenvironment, including time spent in commuting and at home, work or school locations. A summary of UFPs concentrations segregated by MEs and time use for the whole study population is listed in Table 2. The ME where people spent the majority of their time was the indoor environment, with the highest contribution to the daily exposure deriving from residential indoor environments (49%–78%). Time spent outdoors and commuting was generally limited (0%–17% and 0%–8%, respectively). However, some transport microenvironments may represent an important component of human exposure. Commuting time was mostly spent on active (walk/bike: 2%–8%) or motorized transport (car: 2%–7%), both of which represent MEs with high UFP mean concentrations (Table 2).

**Table 1:** Subpopulations defined as a function of gender and segregated by employment status, educational level or age.

Population Characteristic	Characteristic Subgroup	
Gender	Male	Female
Employment Status	Employed	
	Retired **	
	Housewife *	
	Self-employed	
	Student	
Educational Level	0–9 years	
	10–13years	
	14–16years	
	≥17years	
Age	25–34 years	
	35–44 years	
	45–54 years	
	55–59 years	
-	Mean subject	

Notes: * only Female subjects; ** only Male subjects.

**Table 2:** Microenvironments used for the exposure simulation, total sampling time (h), UFP environmental concentrations for summer and winter (mean, standard deviation) (particle/cm^3^), and time-activity patterns (daily percentage of the typical working day).

Category of ME	ME	UFP concentration (pt/cm^3^) ^a,b^						Time Use ^c^ (daily%)		
		Summer			Winter					
		Total sampling time (h)	Mean	S.D.	Total sampling time (h)	Mean	S.D.	Mean	Min	Max
“In-transit” **^a^**	Bike/Walk	11.2	32,214	31,679	9.7	60,277	47,588	2.9	2.0	8.0
	Bus/Tram	6.1	36,798	30,207	6.8	52,386	23,821	1.1	0.0	2.0
	Car/Taxi	10.8	27,034	29,966	10.2	82,890	53,130	3.8	2.0	7.0
	Motorbike/Scooter *	2.8	12,016	7898	2.8	12,016	7898	0.3	0.0	1.0
	Train/Metro	4.5	15,730	9126	2.2	30,643	13,272	0.7	0.0	1.0
Indoor **^b^**	Home Indoor	1705.8	21,645	21,986	1736.5	29,347	29,369	58.0	49.0	78.0
	Work Indoor	57.3	8849	3917	46.8	13,865	6364	0.5	0.0	7.0
	Other Indoor	16.2	25,694	31,743	24.1	22,148	15,309	23.2	1.0	31.0
Outdoor	Other Outdoor **^a^**	10.0	21,008	19,847	10.3	32,219	24,508	1.2	0.0	7.0
	Home Outdoor **^b^**	34.7	12,722	8820	5.6	23,042	15,917	6.8	4.0	17.0
	Work Outdoor **^b^**	6.7	18,716	18,502	8.4	18,880	11,524	1.4	0.0	3.0

Notes: **^a^** [29]; **^b^** PM-CARE Project [30]; **^c^** Source: EXPOLIS project [13,28]; * indirect estimation.

### 2.2. Input Data: Microenvironmental Concentrations

Microenvironmental concentrations (Table 2) were derived from previous studies performed in the city of Milan. UFP concentration for “In-transit” MEs and “Work-Indoor” ME were obtained from a study performed within the central area of Milan in different seasons (from summer 2008 to winter 2009), in which experimental data were collected continuously during each monitoring period along an established urban pathway, moving through different MEs [29]. These data were then updated with an up-to-date measurement, performed following the same study design in 2013 for a total amount of about more than 100 h of performed measurements, distributed in 28 days. The UFP concentrations for the remaining MEs were obtained from a study (PM-CARE project) involving 81 non-smoking senior volunteers living in the urban and suburban area of Milan. During the PM-CARE project, 162 24-hour monitoring sessions were performed in the warm and cold seasons of 2005–2006 following the same sampling protocol and study design [30]. In all these studies a time-activity diary was completed in order to accurately define the concentration data as a function of the different monitored activity and environments. Particle number concentrations (PNC) of airborne UFP were measured using a condensation particle counter (CPC) capable to provide real-time measurement of particles. Data were collected with high sampling frequency (30 s); the instruments were placed in a backpack and carried by one investigator [19] or in a mobile monitoring unit (MMU) developed to sample simultaneously some urban pollutants of interest for public health purposes [30,31]. Since the sampling inlets were not placed in strict correspondence with the breathing zone (the hemisphere of 30 cm radius extending in front of the face) [32], the results refer to the so-called “individual exposure” (in proximity of subjects, within 3 m). The individual exposure approach allows the determination of concentrations without losing accuracy with respect to personal measurements performed in the breathing zone, except for coarse particles [33]. The number concentration metric was selected because of its better accuracy in the continuous monitoring of spatial and temporal variations of UFP concentration, compared with continuous photometric measurement of mass concentrations (especially if these latter are not properly corrected using simultaneous gravimetric data) [24]. Before analysis, data cleaning was performed to exclude invalid values and clear up missing data.

### 2.3. Exposure Model

A microenvironmental probabilistic exposure model was used to simulate the daily personal exposures of urban subpopulations by combining the UFP concentration in selected MEs and the time spent by people in those MEs. In each ME a homogeneous UFP concentration is assumed. The choice for this kind of model (basic microenvironmental model, using a stochastic approach) has been defined due to some considerations. The first was the availability of information on time-activity patterns and environmental concentrations, as this kind of model needs real data in the model-building process. The second consideration was the usability of the model’s output: statistical models are considered to be useful for descriptive analysis and hypothesis testing [5], and thus they are well-suited for this study.

In this work, the combination of the UFP concentration in an ME and the time spent by a subpopulation in the ME was described by Equation (1) and implemented in a Microsoft Excel workbook. An Excel add-on software package was needed to supply the probabilistic functions for the stochastic functionality; a Monte Carlo simulation approach with Latin hypercube sampling (2000 iterations) was chosen for calculation. A probability distribution function is assumed for each parameter: time-activity data were fitted on beta distribution (alpha and beta parameters were calculated starting from the mean and standard deviation of time spent in each ME), while UFP concentrations were fitted on lognormal distributions (calculated again from the means and standard deviations) with Monte Carlo sampling (2000 iterations). From these simulated distributions, random values were then taken using the Latin hypercube method. The sampled parameters were combined to result in a partial exposure for each ME. By summing the partial exposures for each individual ME, the total exposure distributions (“in-transit”, “indoor”, “outdoor” and “total” exposure) was calculated for each considered subpopulations and both for summer and winter. Statistical analysis was performed to identify statistically significant differences (*p* < 0.01) via IBM SPSS Statistics 20.0 (IBM, Armonk, NY, USA), which consisted of factorial analysis of variance (ANOVA) with Helmert contrast and Turkey post-hoc test. All results refer to the daily (24-hour weighted average) mean exposure, using number of particle for cubic centimeter (pt/cm^3^) as the unit.

## 3. Results and Discussion

The seasonal trends of exposure to UFP of different subpopulations living within the city of Milan during a typical working day were estimated using the stochastic microenvironmental model described above. Based on the characteristics reported in Table 1, a total of 26 subpopulations were identified. For each of them an exposure simulation was performed, both for summer and winter, thus generating 52 exposure profiles. A factorial ANOVA was conducted to explore the impact of season, gender and population characteristic on the simulated UFP exposure levels. Subjects were divided into groups according to their gender and consequently age, employment status or educational level, then two seasonal patterns (summer and winter) were defined for exposure simulations. On average, statistically significant differences (*p* < 0.05) were found in total UFP exposure as a function of season and subgroup characteristics (age, employment, education) but not as a function of gender (*p* = 0.0671). Differences were also found among exposures simulated in the studied MEs. On average, the total daily exposure indoors was characterized by the same statistically significant differences in relation to the same variables, but with a better statistical significance between genders (*p* = 0.066). In contrast, there were no statistically significant differences in outdoor total exposures as a function of season (*p* = 0.088) and of population characteristics such as age, employment or education (*p* = 0.905).

### 3.1. UFP Concentrations and Exposure in Urban Microenvironments

The UFP concentrations in Milan have been widely investigated, and seasonal trends, chemical compositions and sources have been described [26,29,34,35,36,37,38]. The measured UFP concentrations of various indoor and outdoor MEs demonstrate a significant variability among indoor MEs and relative homogeneity in outdoor MEs [29]: the highest urban UFP concentrations generally occur while moving along busy streets or in their immediate environments, either on foot or by motorized vehicles and the lowest concentrations are usually detected in indoor environment and in urban green areas. This is consistent with a previous study [39], which states that personal exposure to PM levels were similar between bicycle, bus, and car, while the underground rail tube showed higher concentrations; cyclists were the group with slightly lower exposure, which was influenced by the cyclists’ position on the street and the ability to avoid traffic jams. Regarding the temporal variation of environmental UFP concentrations, appreciable differences were found between working and non-working days, between different periods of the day and between seasons [26,29].

### 3.2. Simulated Exposure

The results of the exposure simulations, segregated by each ME, are shown in Table 3. Results are shown as statistics calculated among all the study subpopulations within the Milan urban area. The following findings were obtained from the analysis of the exposure concentrations in different MEs: highest median exposure (19,561 pt/cm^3^; 80.6% of the total exposure) was obtained, as expected, for indoor environments (Home, Work, Other), which was one order of magnitude higher than the outdoor exposure (651 pt/cm^3^; 2.6%) and well above the exposure simulated for the whole “in-transit” environments (4217 pt/cm^3^; 16.3%). Despite the highest UFP personal concentrations occurred in traffic ME (Table 2), the simulated exposures were actually dominated by indoor environments as (I) the time spent in these environments is very high (Table 2) and (II) the residential environment in Italy is characterized by specific sources, among which the most important is gas cooking [14]. A previous study estimated a daily average UFP exposure of about 16 × 10^4^ pt/cm^3^ for people commuting in Milan, with indoor home exposure providing about 46% of total daily exposure, indoor office exposure about 30%, and transport environments about 24% (almost insensitive to transportation mode) [25]. The results from the present study are up to 20% higher, but in the same order of magnitude, with an average exposure (among all the profiles) of 2.4 × 10^4^ ± 4.65 × 10^3^ pt/cm^3^, with indoor home exposure providing 61.9% ± 5.4% of total daily exposure, indoor office exposure about 11.1% ± 4.1%, and transport environments about 16.7% ± 4.4% (but sensitive to transportation mode). The in-transit MEs show a significant contribution to the total exposure, especially considering the limited amount of time spent in these MEs (8%–13%). Among these, Car/Taxi (1783 pt/cm^3^; 7.5%) and Walk/Bike (1230 pt/cm^3^; 5%) recorded the highest simulated median exposure. On the contrary, the lowest exposure were obtained for Motorbike/Scooter and Train/Metro MEs, which are also characterized by the worst temporal representativeness of collected data (Table 2). Finally, time spent in outdoor MEs did not show a significant contribution to the total estimated exposure (651 pt/cm^3^; 2.6%). The differences in the calculated exposures for each ME were statistically significant (*p* < 0.01) (Kruskal-Wallis one-way ANOVA). Thus, the exposure levels were highly dependent on the spatial behavior and the surrounding microenvironment conditions.

**Table 3:** Summary of the mean simulated exposures segregated for each ME. Results are calculated among all subpopulations within the Milan city area (SD = standard deviation, Min = minimum, Max = maximum, p5 = 5th percentile, p95 = 95th percentile). All results are expressed in particle/cm^3^ (pt/cc). Mean value are expressed also as percentual contribution to total daily exposure (%).

Microenvironment	Mean	Mean (%)	SD	Min	p5	Median	p95	Max
**Individual MEs**								
Bike/Walk	1368	5.5	736	591	668	1230	2637	4874
Car/Taxi	2112	8.3	1350	561	650	1783	4653	5510
Motorbike/Scooter	37	0.4	42	0	0	21	114	123
Train/Metro	163	0.7	91	0	25	140	318	390
Home (Indoor)	14,786	61.9	2862	10,656	11,294	15,098	19,225	22,948
Home (Outdoor)	109	0.4	244	0	0	57	239	1530
Work (Indoor)	2639	11.1	1121	49	142	2600	4194	4301
Work (Outdoor)	267	1.2	345	0	0	164	1057	1373
Other (Indoor)	1629	7.1	652	951	987	1419	2640	4230
Other (Outdoor)	372	1.5	200	0	88	349	745	1031
**Cumulative MEs**								
Outdoor	748	3.1	458	0	237	651	1603	2561
In-Transit	4184	16.7	1790	2121	2240	4217	6987	8012
Indoor	19055	80.1	2974	14,470	15,176	19,561	23,346	25,377
Total	23987	100.0	4650	18,334	18,479	24,604	30,038	32,730

### 3.3. Seasonal Patterns

**Figure 1:**
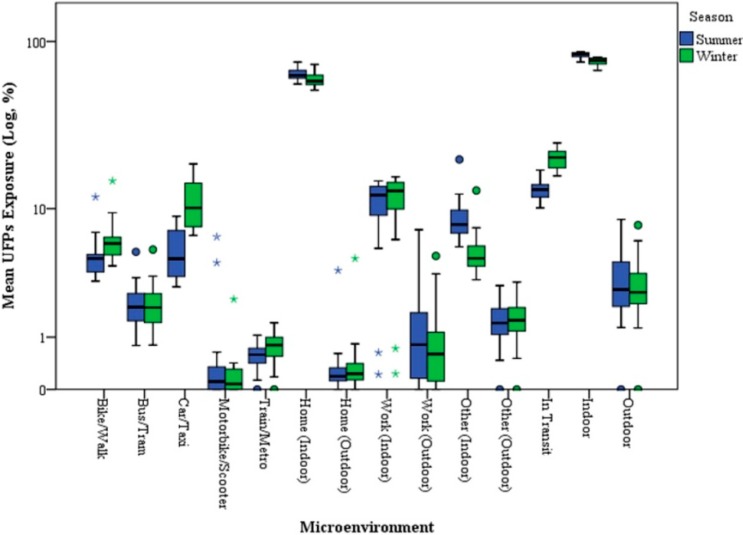
Box plots of calculated UFP exposures (%) estimated in indoor and outdoor microenvironments for summer and winter. The central box comprises values between the 25th and 75th percentiles, and the whiskers show the range of values that falls within 1.5 times the interquartile range beyond the box.

**Figure 2:**
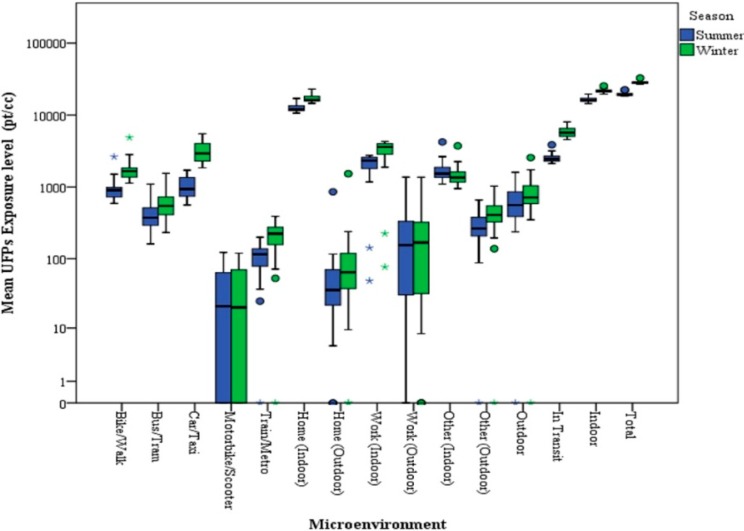
Box plots of calculated UFP exposures (pt/cm^3^) estimated in indoor and outdoor microenvironments for summer and winter. The central box comprises values between the 25th and 75th percentiles, and the whiskers show the range of values that falls within 1.5 times the interquartile range beyond the box.

Published studies for Milan showed a strong seasonal effect in particle concentration values, mainly due to the differences in average dispersion conditions of the atmosphere in summer and winter. Particle concentrations were strongly influenced by seasonal variability, which is more evident for the finer particle sizes, with higher values in winter [35,36,37]. This seasonal variation is, essentially, linked more to the differences in average thermodynamic and meteorological conditions of the atmosphere than to the variations in the type or number of emitting sources. However, the observed seasonal behavior of particulate concentrations may also be ascribed to the presence of additional emission sources (*i.e.*, domestic heating) during the cold season, which contributes to primary as well as to secondary aerosol production because of the large emission of gaseous precursors. The simulated exposures, segregated by ME and season, are shown in Figure 1 and Figure 2. Here, a significant difference (*p* < 0.01; Mann–Whitney U Test) was observed between the exposures experienced in the two different seasons: on the whole, the highest median exposure (28,415 pt/cm^3^) was obtained in winter (W), as expected; this level was about 45% higher than in summer (S) (19,558 pt/cm^3^). Similarly, the average UFP exposure experienced by 24 Italian couples was higher in winter (women: 2.9 × 10^4^; men: 1.3 × 10^4^ part/cm^3^) than summer (women: 1.8 × 10^4^; men: 9.2 × 10^3^ part/cm^3^) [11]. The whole indoor exposure showed a significant increase in median values from summer to winter (16,041 pt/cm^3^ and 21,511 pt/cm^3^, respectively), but this is accompanied by a slight decrease in the relative contribution of indoor environments to the total exposure (S: 84.4%, W: 77.5%). The exposure levels calculated for outdoor environments appear to be almost unchanged between the seasons (S: 561 pt/cm^3^; W: 716 pt/cm^3^) as well as their contribution to the total exposure (S: 2.8%; W: 2.6%). Contrarily, transit MEs show a strong variation between seasonal simulations: in-transit median exposure in winter (5690 pt/cm^3^) was about 130% higher than in summer (2459 pt/cm^3^). Moreover, the relative contribution of in-transit exposure to the total daily exposure increases from a median value of 13.2% in summer to 20.7%. The Car/Taxi and Walk/Bike MEs recorded the highest increases in partial exposure simulation. The lowest exposure was obtained for Motorbike/Scooter and Train/Metro MEs.

### 3.4. Subpopulation Characteristics: Gender

The UFP exposure simulations were made on the basis of the representative time pattern profile determined for males and females. Results separated by season and gender show a moderate difference between the total exposures of male (M) and female (F) subpopulations (Table 4). This is consistent with previous studies on exposure to submicrometer particles [11,17], which estimated that females receive higher daily doses than males. This difference should be not addressed to a “gender effect”, but can be explained through the different lifestyle between female and male. According to the time use data, differences were found for the separate MEs: the mean total indoor exposure represents again the highest fraction of the total estimated exposure, which is stably higher for women (S: 16,634 pt/cm^3^; W: 22,121 pt/cm^3^) than for men (S: 15,464 pt/cm^3^; W: 20,789 pt/cm^3^). Mean contributions of in-transit exposure to the total exposure were higher for men (S: 2567 pt/cm^3^; W: 6119 pt/cm^3^) than for women (S: 2347 pt/cm^3^; W: 4929 pt/cm^3^), as well as the simulated outdoor exposures. After the indoor MEs, the major mean contribution to the total exposure was found in the in-transit MEs: the Car/Taxi and Walk/Bike MEs recorded the highest modeled exposures. Finally, the lowest mean exposures were obtained for Motorbike/Scooter, Train/Metro and “Other Outdoor” MEs.

### 3.5. Subpopulation Characteristic: Age

UFP exposure simulations were made for 4 different age-stratified subpopulations. The results, separated by season, are shown in Table 5. The in-transit and indoor MEs show a significant variation among subpopulations: indoor exposure ranged from 76.1% (in winter) to 85.2% (in summer) of total estimated exposure, while the relative contribution of in-transit exposure was about 12% in summer and 20% in winter, as discussed above. Simulated outdoor exposures were almost unchanged with age, representing always a small fraction of the total exposure (2.4%–3.6%). The highest mean indoor exposure was observed for 55–59 years old people, both in summer (16,823 pt/cm^3^) and in winter (22,361 pt/cm^3^). This could explain the fact that this subpopulation was characterized by the highest mean total exposure, too (S: 19,933 pt/cm^3^; W: 29,064 pt/cm^3^). In contrast, the highest mean in-transit exposure was observed for the youngest population (25–34 years), both in summer (2567 pt/cm^3^) and in winter (5860 pt/cm^3^), as a direct consequence of the major amount of time spent in this kind of ME, in which the highest UFP concentrations were found. In fact the “age effect” may be ascribable to the different lifestyles, since other factors (characteristics of the different age groups and performed activities) were found to have negligible effect on daily doses of submicrometer particles [12,16,17]. Thus, the reason of the outlined differences between subgroups (and in comparison with previous studies) may be found in the different particle exposure levels experienced in different MEs.

**Table 4:** Summary of the mean simulated exposures segregated for each ME and season. Results are shown for the mean male and female profiles among all subpopulations.

ME	Mean Subpopolation Exposure (Male)								Mean Subpopolation Exposure (Female)							
	Time use (fraction)		Summer			Winter			Time use		Summer			Winter		
	Mean	SD	Mean (%)	Mean (pt/cm^3^)	SD (pt/cm^3^)	Mean (%)	Mean (pt/cm^3^)	SD (pt/cm^3^)	Mean	SD	Mean (%)	Mean (pt/cm^3^)	SD (pt/cm^3^)	Mean (%)	Mean (pt/cm^3^)	SD (pt/cm^3^)
***Individual***																
Bike/Walk	0.02	0.03	3.8	706	1329	4.8	1324	2221	0.03	0.02	5.1	993	1530	6.5	1791	2143
Bus/Tram	0.01	0.02	1.8	345	871	1.7	477	1020	0.01	0.02	2.5	478	864	2.6	713	1331
Car/Taxi	0.05	0.05	7.1	1330	2873	14.4	4027	5523	0.03	0.03	3.7	713	1339	7.7	2134	3064
Motorbike/Scooter	0.01	0.02	1.8	74	252	7.1	70	213	0.00	0.01	0.1	21	119	0.1	25	214
Train/Metro	0.01	0.02	06	112	290	0.8	221	515	0.01	0.02	0.7	140	318	1.0	265	539
Home Indoor	0.53	0.08	61.2	11,502	12,541	55.9	15,581	15,996	0.59	0.12	66.4	12,866	13,271	62.5	17,273	17,293
Home Outdoor	0.00	0.01	0.3	61	206	0.4	103	342	0.00	0.01	0.2	43	138	0.3	75	221
Work Indoor	0.29	0.11	13.5	2534	1551	14.2	3962	2504	0.24	0.13	11.2	2160	1637	12.3	3404	2666
Work Outdoor	0.02	0.06	2.3	430	1432	1.6	446	1321	0.00	0.02	0.4	76	338	0.3	79	279
Other Indoor	0.06	0.06	7.6	1428	2888	4.5	1247	1791	0.06	0.06	8.3	1608	2690	5.2	1444	1975
Other Outdoor	0.01	0.03	1.5	284	772	1.6	440	1213	0.01	0.02	1.4	265	569	1.5	420	837
***Cumulative***																
Outdoor	0.04	0.10	4.1	775	1667	3.5	988	1842	0.02	0.13	2.0	383	676	2.1	574	915
In-Transit	0.09	0.13	13.7	2567	3311	21.9	6119	6067	0.08	0.08	12.1	2347	2238	17.8	4929	3963
Indoor	0.87	0.25	82.2	15,464	12,992	74.5	20,789	16,318	0.90	0.27	85.9	16,634	13,627	80.1	22,121	17,546
Total	1.00	0.01	100.0	18,806	13,395	100.0	27,897	17,453	1.00	0.01	100.0	19,363	13,820	100.0	27,623	18,055

**Table 5:** Summary of the mean simulated exposures, segregated for cumulative MEs and seasons. Results are shown for age-stratified subpopulations.

Subpopulation (age)	ME (cumulative)	Summer					Winter				
		Time use (fraction)		Exposure			Time		Exposure		
		Mean	SD	Mean (%)	Mean (pt/cm^3^)	SD (pt/cm^3^)	Mean	SD	Mean (%)	Mean (pt/cm^3^)	SD (pt/cm^3^)
25–34 years	Outdoor	0.03	0.01	3.2	610	344	0.03	0.01	2.8	795	359
	In-Transit	0.10	0.01	13.5	2567	148	0.10	0.01	21.1	5860	823
	Indoor	0.88	0.03	83.3	15,820	61	0.88	0.03	76.1	21,113	308
	Total			100.0	18,997	431			100.0	27,768	874
35–44 years	Outdoor	0.04	0.02	3.6	679	295	0.04	0.02	2.7	750	183
	In-Transit	0.08	0.01	12.1	2282	229	0.08	0.01	19.4	5307	1040
	Indoor	0.89	0.02	84.3	15,935	1167	0.89	0.02	77.9	21,385	1481
	Total			100.0	18,896	642			100.0	27,442	258
45–54 years	Outdoor	0.03	0.01	2.4	444	175	0.03	0.01	2.5	692	247
	In-Transit	0.09	0.01	12.4	2364	92	0.09	0.01	19.4	5408	642
	Indoor	0.89	0.01	85.2	16,244	1283	0.89	0.01	78.2	21,856	1347
	Total			100.0	19,053	1016			100.0	27,956	458
55–59 years	Outdoor	0.04	0.02	3.3	644	309	0.04	0.02	3.0	858	271
	In-Transit	0.09	0.02	12.4	2466	463	0.09	0.02	20.1	5846	1050
	Indoor	0.89	0.04	84.3	16,823	1445	0.89	0.04	76.9	22,361	1392
	Total			100.0	19,933	673			100.0	29,064	72

### 3.6. Subpopulation Characteristics: Employment Status

UFP exposure simulations were also made for five subpopulations of different employment status. The results (Table 6) showed a significant variation among these subpopulations: the total indoor exposure represented the highest fraction of the total estimated exposure, but ranging in a wide interval (67.7%–84.7%). The contributions of in-transit exposure to the total exposure were also found to be quite variable (10.1%–24.5%), as well as the simulated exposure in outdoor environments, where a relevant fraction of the total exposure was also estimated for some profiles (up to 7.8%). The highest mean indoor exposures were observed, both in summer and winter, for housewives (S: 19,564 pt/cm^3^; W: 25,377 pt/cm^3^) and students (S: 17,954 pt/cm^3^; W: 22,791 pt/cm^3^). In contrast, the highest mean commuting exposures were observed, both in summer (3862 pt/cm^3^) and winter (8012 pt/cm^3^), for retired people, as well as for their total outdoor exposure (S: 1516 pt/cm^3^; W: 2561 pt/cm^3^). Thus, once again, differences between people can be explained by the time-activity pattern of the individuals, as well as the environments in which they spend their time. In fact, people can experience different exposure profiles and short-term exposures that may contribute significantly to daily average exposure: recently it has been found that the average exposure to UFP experienced by Italian homemakers were higher (roughly twice) than their spouses (full-time workers) [11].

### 3.7. Subpopulation Characteristic: Educational Level

Table 7 shows the UFP exposure estimates for four different subpopulations segregated by their educational level (years of scholarity). As discussed above, all winter exposures were typically higher than summer exposures. The total indoor exposure represents the highest fraction of the total estimated exposure, ranging in a limited interval (74.7%–81.6 %). The contributions of in-transit exposure to the total exposure were also found to be quite variable (12.4%–21.7%), as well as simulated exposure for outdoors (2.0%–5.3%). The highest mean indoor exposures were observed, both in summer and winter, for the categories with higher educational level (“10–14 years” and “≥17 years”), which also experience the highest total exposure. Again, the highest mean in-transit exposure was found for the category “10–14 years”, while for the outdoor MEs, the highest mean exposures was found for the category “0–9 years”.

**Table 6:** Summary of the mean simulated exposures, segregated for cumulative MEs and season. Results are shown for employment-segregated subpopulations within the Milan urban area.

Subpopulation (employment status)	ME (cumulative)	Summer					Winter				
		Time use (fraction)		Exposure			Time		Exposure		
		Mean	SD	Mean (%)	Mean (pt/cm^3^)	SD (pt/cm^3^)	Mean	SD	Mean (%)	Mean (pt/cm^3^)	SD (pt/cm^3^)
Student	Outdoor	0.02	0.03	1.9	402	569	0.02	0.03	1.7	495	700
	In-Transit	0.10	0.02	13.5	2868	431	0.10	0.02	20.7	6100	802
	Indoor	0.90	0.01	84.6	17,954	392	0.90	0.01	77.6	22,791	819
	Total			100.0	21,224	254			100.0	29,386	922
Employed	Outdoor	0.03	0.01	2.5	469	133	0.03	0.01	2.3	634	111
	In-Transit	0.09	0.01	12.8	2383	165	0.09	0.01	19.7	5317	776
	Indoor	0.89	0.01	84.7	15,752	472	0.89	0.01	78.0	21,090	578
	Total			100.0	18,603	175			100.0	27,041	309
Self-employed	Outdoor	0.04	0.04	3.8	721	684	0.04	0.04	2.9	847	704
	In-Transit	0.10	0.01	13.9	2636	255	0.10	0.01	23.2	6606	1073
	Indoor	0.88	0.03	82.3	15,586	580	0.88	0.03	73.9	20,982	767
	Total			100.0	18,943	359			100.0	28,435	1010
Housewife *	Outdoor	0.03	-	2.5	559	-	0.03	-	2.8	873	-
	In-Transit	0.08	-	10.1	2262	-	0.08	-	16.0	4995	-
	Indoor	0.89	-	87.4	19,564	-	0.89	-	81.2	25,377	-
	Total			100.0	22,386	-			100.0	31,246	-
Retired **	Outdoor	0.10	-	6.8	1516	-	0.10	-	7.8	2561	-
	In-Transit	0.13	-	17.3	3862	-	0.13	-	24.5	8012	-
	Indoor	0.77	-	75.9	16,894	-	0.77	-	67.7	22,157	-
	Total			100.0	22,271	-			100.0	32,730	-

Notes: * only Female subject; ** only Male subjects.

**Table 7:** Summary of the mean simulated exposures, segregated for cumulative MEs and seasons. Results are calculated for subpopulations segregated by educational level (*i.e.*, year of scholarity).

Subpopulation (Educational Level)	ME (cumulative)	Summer					Winter				
		Time use (fraction)		Exposure			Time		Exposure		
		Mean	SD	Mean (%)	Mean (pt/cm^3^)	SD (pt/cm^3^)	Mean	SD	Mean (%)	Mean (pt/cm^3^)	SD (pt/cm^3^)
0–9 years	Outdoor	0.06	0.05	5.3	1001	850	0.06	0.05	4.2	1179	785
	In-Transit	0.09	0.01	13.1	2524	276	0.09	0.01	21.1	6006	1082
	Indoor	0.86	0.06	81.6	15,823	1914	0.86	0.06	74.7	21,288	2447
	Total			100.0	19,348	788			100.0	28,473	581
10–13 years	Outdoor	0.03	0.01	3.5	658	294	0.03	0.01	3.1	853	255
	In-Transit	0.09	0.01	12.9	2431	233	0.09	0.01	19.7	5438	1119
	Indoor	0.89	0.02	83.7	15,841	879	0.89	0.02	77.2	21,274	855
	Total			100.0	18,929	352			100.0	27,565	518
14–16 years	Outdoor	0.03	0.01	3.2	629	251	0.03	0.01	3.0	869	329
	In-Transit	0.10	0.01	13.6	2658	110	0.10	0.01	21.7	6201	1112
	Indoor	0.88	0.02	83.2	16,277	1042	0.88	0.02	75.2	21,428	1174
	Total			100.0	19,563	681			100.0	28,497	267
≥17years	Outdoor	0.02	0.01	2.2	418	217	0.02	0.01	2.0	556	231
	In-Transit	0.09	0.01	12.4	2346	149	0.09	0.01	19.1	5236	397
	Indoor	0.90	0.01	85.4	16,116	518	0.90	0.02	78.8	21,576	856
	Total			100.0	18,880	153			100.0	27,368	228

### 3.8. Discussion

Analysis of literature data on the time spent in the study MEs [28] showed that people used to spend much less time outdoors (about 1% of the day) than indoors (male: 87% ± 25%; female: 90% ± 31%) (Table 4). The time indoors was mostly spent at home, equaling approximately two thirds of all the time spent indoors and more than 50% of the day. People spent on average about 30% of their time at workplace, mostly indoors (male: 29% ± 11%; female: 24% ± 13%). This was true for all subgroups by gender, age, educational level, employment status and season. Women had the highest average time spent indoors, and regarding employment status, self-employed workers and retired people spent the least amount of time indoors. Typically, rather long time periods (with small standard deviations) were spent on average in major MEs such as home indoors or work indoors. In contrast, only short periods (with relatively high standard deviations) are spent outdoors or in-transit. In-transit time represented about 10% of the typical working day (Table 4). Traveling by car and walking or biking are the most popular means of transportation for the adult urban population of Milan. There are also noticeable differences in the average use of some means of transportation. On average, driving a car and walking/biking each account for approximately more than half of the total time spent in-transit. Difference in total time in traffic was found between specific subgroups; gender and employment status are very important factors. Time spent in cars has been shown to be one of the most important determinants of traffic exhaust exposure [40]. Walking or biking on city roads also often results in very close proximity to fresh traffic exhaust. Public transportation in general was more likely to be used among women. Age did not significantly contribute to the time-activity patterns in our study, while the general employment status often affected the time-activity patterns. Men generally spent more time in-transit than women. Employed participants spent more time in-transit than others. The exposure levels show a stronger correlation with time spent in each ME (r_spearman_ 0.952; *p* < 0.01) rather than with the ME’s UFP concentrations (r_spearman_ 0.149; *p* < 0.01), thus the results from the present study showed that the variability in UFP exposure is mainly related to behavioral factors (e.g., mode of transport) and seasonal patterns, both of which have a very large influence on the human exposure to UFP [35,36,37]. Thus, demographic and sociodemographic factors may be considered as the major determinants of UFP exposure in urban environments. The results from this modeling study are consistent with literature [13,14,18]. UFP concentrations in Milan have been widely investigated, and seasonal trends, chemical compositions, and sources have been described [26,29,34,35,36,37,38]. The measured UFP concentrations within a variety of indoor and outdoor MEs demonstrate significant variability among some indoor MEs and relative homogeneity in outdoor MEs [29]: the highest urban UFP concentrations generally occur when moving along busy streets or in their immediate environments, either on foot or by motorized vehicles, and the lowest concentrations are usually detected in indoor environment and in urban green areas. Regarding the temporal variation of environmental UFP concentrations, appreciable differences were found [26,29] between working and non-working days, at different times of the day and between seasons. Previous studies estimated a daily average exposure to UFP of about 16 × 10^4^ pt/cm^3^ for people commuting in Milan, with indoor home exposure accounting for 46% of total daily exposure, indoor office exposure about 30%, and transport environments about 24% (almost insensitive to transportation mode) [25]. The results from the present study have the same order of magnitude, with a higher average estimated exposure (among all profiles) of 24 × 10^4^ ± 4.65 × 10^3^ pt/cm^3^, with indoor home exposure giving 61.9% ± 5.4% of the total daily exposure, indoor office exposure about 11.1% ± 4.1%, and transport environments about 16.7% ± 4.4% (but *sensitive* to transportation mode). The results from the present study confirm that the variability in UFP exposure is also related to behavioral factors (e.g., mode of transport) and seasonal patterns, both of which have a very large influence on human exposure to UFP [41,42,43]. Thus, demographic and sociodemographic factors may be considered as major determinants of UFP exposure in urban environments.

### 3.9. Assumptions and Limits

The following assumptions were included in the model:

(I) The CPC used in these studies (model P-Trak Ultrafine Particle Counter 8525; TSI Inc., Shoreview, MN, USA) can measure particles ranging from 0.02 to 1 μm in size (so UFP data include also particles with dimension >100 nm, although their number concentration is assumed to be very low with respect to those in the 0–100 nm interval) and has shown effectiveness in detecting the variations of PNC in urban environments [26]. As a general concept, for some in-transit MEs involving transient aerosol dynamics (*i.e.*, characterized by very rapid aerosol generation and dilution processes), the measurements should be performed using aerosol measurements with frequencies high enough to track the steep changes that the aerosols undergo, thus a higher temporal resolution would be desirable for in-transit environments [44].

(II) The lognormal distribution was used as the default distribution for UFP concentrations. Despite possible deviancies from lognormality, this assumption could work fine in the current model as environmental pollutant concentrations are often found to follow lognormal distribution [8,11,38]. Moreover, the current model used also fitted beta distributions to describe the time fractions spent in each ME [11]. For one ME (motorbike/scooter) UFP measurement was not available, thus an indirect estimate was made, using outdoor concentrations recorded for sporadic measurement. Note that this approximation introduced only a very limited error in the total exposure estimations, because a very limited time (<1%) were globally spent in this ME.

(III) Model validations were not possible because it was not possible to collect exposure data for the study subpopulations. Since the study examined the exposure distributions among the selected subpopulations, it is almost impossible to conduct a model validation, which should require personal measures for a large population. Thus, the performance of the model simulations strictly depends on the quality of the input data for time-activity pattern and microenvironmental measures. Therefore, it is crucial that these data appropriately reflect the specific subgroup of population under various environmental conditions. This problem has been stressed by many researchers in the field of exposure modeling and it would be very helpful if more databases on environmental concentration data and exposure-relevant time-activity data could be published and made available [15]. Despite this, a previous research [2] showed that the time-activity data used for modeling [28] are a helpful tool for evaluating air pollution exposures in different scenarios, population groups and locations (the model predicted mean population exposure levels in four European cities with an accuracy of >20%) and for helping researchers to understand the factors that affect exposure levels. Further, the concentration input parameters used for the simulations were obtained from previous researches [26,29], which are expected to reflect the microenvironmental concentration under certain conditions (typical weekday, in two season) for the general (non-smoking) population. This approach should provide a sufficient understanding of exposures in urban areas. Still, it is clearly evident that exposure research in the urban microenvironment has numerous inferences and there are various factors that can potentially affect personal exposure concentrations [38], especially when considering particular subpopulations.

(IV) In the absence of validation, however, it is questionable what solution would approach the real exposure situation most accurately. Model uncertainty includes uncertainties in the selection of the distributions, definition of the MEs and modeled activities, selection of averaging times and number of iterations, and generation of the random numbers, and so forth [45,46]. In the basic equation of our model uncertainty is not included. The simplifications used in the selection of microenvironments and the selection of parametric distributions, however, introduce uncertainties to the model structure [11]. Full analysis of the model uncertainty would significantly broaden the focus and volume of this article. Further, the comparison of the modeled and measured exposures is not possible, because this study used a retrospective approach, using old data, incorporating a number of factors that cannot be captured by a single air monitoring campaign nowadays. Therefore, only measurement errors causing parameter uncertainty may be evaluated in the presented models. Thus, a nominal range sensitivity analysis was carried out accordingly. This sensitivity analysis was performed by investigating the effect of parameters on the estimated exposures [46]: the model’s inputs were individually varied across their entire range of plausible values, while holding all other inputs at their base values. The sensitivity was presented as a positive or negative percentage change compared with the base values. Time spent in indoor MEs and the corresponding concentrations were found to be the most important parameter leading to possible prediction errors (±60%). In-transit MEs (car/taxi, bike/walk) were another source of possible uncertainties, as they were affected by high variability (±10% for each ME).

(V) A limitation of this study is the rather small sample size in the definition of time-activity patterns and the use of quite old data [18], with the intrinsic assumption that time-activity patterns were unchanged in the last 15 years. Furthermore, measurements in the urban areas were not carried out simultaneously for practical reasons, but were derived from a previous study of the research group. This may have induced systematic differences in measurements because of temporal factors. The lack of such data was one of the main reasons we conducted this retrospective exposure modeling, incorporating several factors that were not captured by a single air monitoring campaign but strongly influence personal exposures, such as time-activity patterns, residential and workplace measurements.

(VI) This paper describes the simulation of daily mean exposure for different subpopulations in indoor and outdoor MEs. Potential bias may occur when considering the source data and in particular, the temporal representativity of the summer and winter seasons, the representativity of the considered MEs and the representativity of the presence of indoor and outdoor sources during the measurements. Although the reference studies [29,30] have considered temporal and spatial variability for the studied MEs, the design and methods imply some limitations in the generalizability of these findings. For example, although the MEs were chosen to reflect common urban activities and general trends, the specific locations were selected according to a systematic and technical protocol. Thus, these MEs could not be representative of the average concentrations in the same kind of MEs across Milan or in other cities. The UFP residential and indoor concentrations were mainly collected within the Milan urban and suburban area, involving a quite large number of volunteers (N = 81) for a wide monitoring period (N = 162 days, >3800 h). Thus, the results from the monitoring campaigns are assumed to reflect common residential activities and general trends, but it must be considered that a potential bias may occur when ignoring specific variability factors. On the contrary, the motorbike/scooter and train/metro MEs were investigated for a limited time (few hours on the whole, see Table 2), and the corresponding measurements of UFP concentrations cannot be considered necessarily representative of the general exposure scenario occurring in these specific in-transit conditions.

## 4. Conclusions

In this work, a microenvironmental stochastic simulation model was used in order to simulate the average daily ultrafine particles exposure of adult subpopulations (defined on the basis of gender, age, employment status and educational level) in a major Italian urban area and in different exposure scenarios (typical working day in summer and winter). Although the number of profiles taken for this study is too small to yield statistical evidence, some general conclusions can be drawn and this study provide seasonal information on the average exposure to UFP in various microenvironments for a wide range of subpopulations. The estimated average daily exposure was higher in winter than in summer. The highest median exposures were obtained, as expected, for indoor environments, which were one order of magnitude higher than outdoor exposures and well above the simulated commuting exposures. The in-transit MEs contributed significantly to the total daily exposure, mostly considering the limited amount of time spent in these MEs. The Car/Taxi and Walk/Bike MEs were characterized by the highest simulated median exposures. The outdoor MEs did not show an important contribution to the total estimated exposure. Total daily exposure simulations also showed a moderate difference between genders; differences between genders were also found in some specific MEs. The mean total indoor exposure, which represented the highest fraction of the total estimated exposure, was stably higher for women than for men. In contrast, the mean contribution of commuting (in-transit) and outdoor environments to the total daily exposure was higher for men. Thus, demographic and sociodemographic factors, as well as environmental patterns, have to be considered as major determinants of pollutant exposure in urban environments. Large-scale experiments including personal measurements might help to improve modeling approaches for a better estimation of actual exposure on a statistically sound basis.
